# Morphology and performance of polyvinyl chloride membrane modified with Pluronic F127

**DOI:** 10.12688/f1000research.15077.2

**Published:** 2018-07-10

**Authors:** Nasrul Arahman, Afrilia Fahrina, Mukramah Yusuf Wahab, Umi Fathanah

**Affiliations:** 1 Department of Chemical Engineering, Syiah Kuala University, Banda Aceh, Indonesia

**Keywords:** polyvinyl chloride (PVC), Pluronic F127, pore forming agent

## Abstract

**Background:** Attempts to modify the morphology of membrane for application in industrial separation are being undertaken by many researchers. The present study discusses the morphological modification of polyvinyl chloride (PVC) membrane by combining the hydrophilic surfactant Pluronic F127 (PF127) in a polymer solution to improve the performance of the membrane.

**Method:** The membrane is formed using the non-solvent induced-phase separation (NIPS) method. PF127 is added to the membrane solution as a membrane modifying agent. The effects of the surfactant concentration in the dope solution on the permeability of pure water, solute rejection, hydrophilic characteristics, and membrane morphology are investigated.

**Results:** Higher concentrations of PF127 had a significant effect on the permeability of pure water. The highest membrane permeation was 45.65 l/m ^2^.hr.atm with the addition of 7% PF127 additive.

**Conclusion:** PF127 is successfully proposed as a membrane pore-forming agent in this work; the blending of this additive in appropriate amounts in the polymer solution is adequate to improve the performance of the PVC membrane.

## Introduction

Nowadays, separation of contaminant elements from drinking water using membrane technology is developing rapidly. Membrane separation technology has been adopted in many industries, owing to its numerous advantages compared with other common methods. One of the most widely applicable membrane separation technologies in industry is the use of a group of ultrafiltration (UF) membranes, particularly for the process of water purification ^1, 2^. In view of the requirements for application in the water treatment industry, the modification of UF membrane to generate high flux, improve the resistance to fouling and chemical substances, and provide good mechanical properties is being continuously improved ^3, 4^.

**Figure 1.**
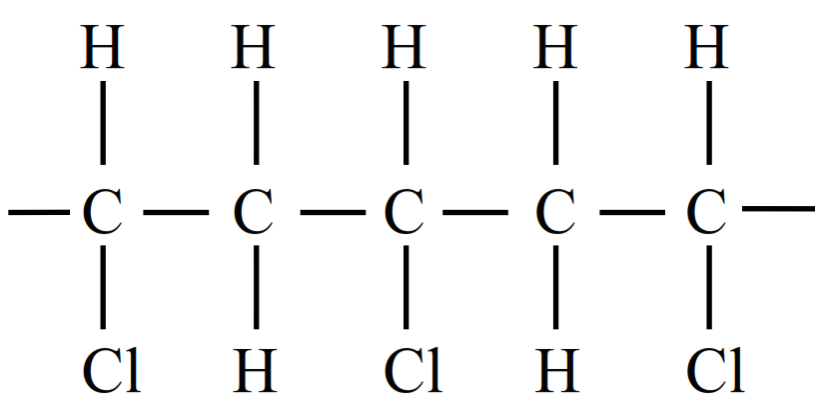
The molecular formula of polyvinyl chloride.

Polyvinyl chloride (PVC), with the molecular formula shown in Figure 1, is a relatively cheap polymer with suitable chemical characteristics for use as a membrane material. Hydrophobic PVC polymers cause fouling of the membrane pores due to the adherence of organic molecules to the surface of the membrane. Numerous methods have been developed to improve fouling resistance. The most common method is improving the hydrophilicity of the membrane material to minimize the attachment of foulant molecules ^5, 6^.

The hydrophilic polymers that are frequently used as an additive are polyvinylpyrrolidone, polyethylene glycol, Brij, Tetronic, and Pluronic ^7, 8^. Of these, Pluronic is used as a surface modifying agent for many hydrophobic polymers. Raslan and Mohammad ^9^ added Pluronic F127 to a polysulfone membrane. The resulting membrane is resistant to fouling and possesses good pore distribution ^9^. Pluronic has also been used to improve the anti-fouling of cellulose acetate (CA) membranes ^10^. The combination of CA polymer and Pluronic surfactant results in a membrane that is more resistant to fouling and has a more stable filtration profile. Another study investigate the hydrophilic surfactant Tween20 and Tween80 to enhance the permeation and antifouling properties of PVC membrane ^11^. The surfactants were added as 0; 1; 3; 5; and 7 % to dope membrane. The result showed that higher concentration of the both surfactant resulting higher water flux. The highest water flux is 328,6 L/m2.h with 7% Tween20 addition. Contrary to the rejection performance of BSA, the result showed decreasing rejection value in higher surfactant addition. The BSA rejection by PVC original membrane is about 97%, after addition of surfactant additives the rejection value decreased up to around 86-87,5% at 7% Tween20 and Tween80.

**Figure 2.**
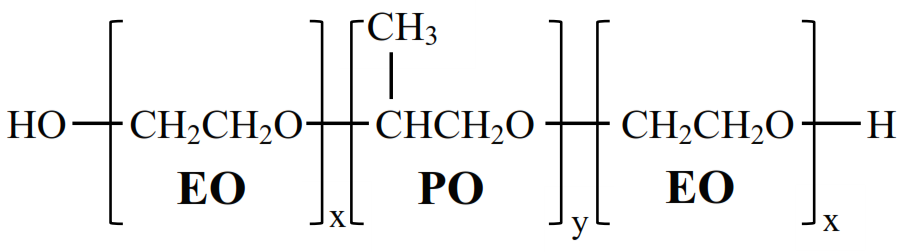
The molecular formula of PF127. x, ethylene oxide (EO) number, y, propylene oxide (PO) number.

In this study, pluronic was developed to improve the performance of PVC membranes. PF127 is a copolymer with two segments—hydrophilic and hydrophobic ( Figure 2). The polyethylene oxide (PEO) segment of PF127 improves the hydrophilic characteristics of the membrane’s surface, while polypropylene oxide, which is hydrophobic, attaches closely to the matrix of the membrane ^9^. The hydrophile–lipophile balance value of PF127 ranges from 18 to 23 ^12^. In this study, PF127 is used as an additive to improve the performance of a PVC membrane.

## Methods

### Materials

Polyvinyl chloride (PVC) with an average molecule weight of 43,000 Da was obtained from Sigma-Aldrich (Merck KGaA, Darmstadt, Germany). Dimethyl acetate (DMAc) solvent was obtained from Wako, Pure Industries Japan. Distilled water was produced in the laboratory. PF127 was obtained from BASF Co. (Ludwigshafen, Germany). Dextran with an average molecular weight of 10,000 Da, which was used for the rejection test, was bought from Sigma-Aldrich. All chemicals were used directly without previous treatment.

### Membrane preparation

The wet inversion technique was adopted to prepare the membrane using water as a non-solvent coagulation media. PVC with a concentration of 15 wt% and PF127 with concentration of 1, 3, 5 and 7 wt% were dissolved in DMAc to improve the performance of the membrane The solution was stirred with a magnetic stirrer at 200 rpm until it was homogeneous. The homogeneous membrane solution was left for 24 hours at room temperature to completely discharge the air bubbles. The solution was then framed on a glass plate using an automatically adjustable applicator (YBA-3, Yoshimitsu, Japan) at a thickness of 450 µm. The glass plate containing membrane was dipped in a coagulation bath of distilled water. The de-mixing process between the DMAc solvent and non-solvent distilled water solidified the membrane and separated it from the glass plate.

### Membrane morphology

Membrane morphology was observed using scanning electron microscopy (SEM) (Hitachi Co, S-800) with an accelerating voltage of 15 kV. To obtain a clean and dry sample, about 1 cm ^2^ of the membrane sample was freeze-dried (Eyela FD-1000, Japan) for 24 hours. To ensure that the structure of the membrane was not damaged, the membrane sample was ruptured in liquid nitrogen. Next, the membrane sample was mounted on the metal module, followed by the coating process with Pt/Pd sputtering. The coated sample was inserted into the SEM apparatus, and the photo was captured at 5.0 kV. Three images was collected for each Three images each were collected for PVC membranes containing 0, 3, 5 and 7% P127.

### Permeation experiment

The permeability of water and solute rejection were tested with the module of dead-end filtration (Advantec, UHP-43K, Japan). The transmembrane pressure was regulated at a pressure of 0.5 atm. The effective membrane surface area that passed by water was 0.023 m ^2^. The testing of water permeability was conducted four times, and the average values was taken to determine the final permeability. The permeability coefficient of pure water was counted using Equation 1.

$${\text{L}}_{\text{p}}=\frac{\text{V}}{\text{A}\times \text{t}\times \Delta_{\text{p}}}\;(1)$$

Where Lp = permeability coefficient (L/m ^2^.jam.atm); V = permeate volume (L); A = membrane surface area (m ^2^); and Δp = pressure change (atm).

A dextran solution of 100 p.p.m. was prepared to analyze the rejection efficiency. Equation 2 was used to calculate the rejection value of the fabricated membrane.

$$\text{R}=\left(\text{1}-\frac{{\text{C}}_{\text{p}}}{{\text{C}}_{\text{f}}}\right)\times 100\;(2)$$

Where R = rejection coefficient; Cp = permeate concentration; and Cf = concentration of feed.

### Membrane hydrophilicity

The hydrophilicity of the surface of the membrane was measured using a water contact angle meter (Kyowa Kaiwenkagaku, Saitama, Japan, CA-A). The contact angle is the angle formed between the surface of the test material and the pure water dropped onto the surface of the membrane ^13^. Each sample was measured 10 times, and the average value of the measurement was the value of the water contact angle of the membrane sample.

### Membrane shrinkage

To study the effect of the blending of pluronic additives on the toughness of the PVC/ PF127 membrane, a membrane shrinkage test was performed. The wet cast of membrane at each PF127 concentration was dried in the oven for 24 hours at 80°C. The shrinkage of the membrane was calculated using Equation (3).

$$\text{Membrane}\;\text{shrink}\;\left(\text{\%}\right)=\frac{L_{0}-L_{1}}{L_{0}}\times 100\;(3)$$

Where L _0_ = length of wet membrane (cm) and L _1_ = length of dried membrane (cm).

## Results and discussion

### Membrane morphology

**Figure 3.**
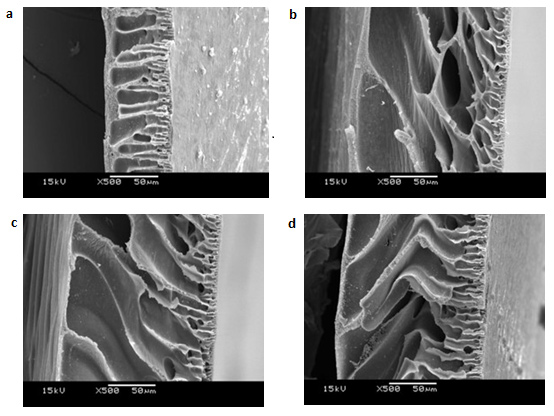
Morphology structure of PVC membrane on transverse part at PF127 concentrations of 0% ( **a**); 3% ( **b**); 5% ( **c**); and 7% ( **d**). Each image is representative of n=3 scanning electron microscopy images.

The results of the SEM analysis of the cross-sections of all the membranes are shown in Figure 3. The transverse part of the original PVC membrane has an asymmetric structure consisting of a thick, dense structure in the top layer and a finger-like structure in the center path of the cross-sectional area. The formation of a membrane structure fabricated using the wet inversion technique happens during coagulation, in which the DMAc solvent is leached out of the matrix of the dope solution and water as a non-solvent diffuses into the membrane. This phenomenon causes the formation of membrane pores and a finger-like macrovoid structure ^14^. The structure of the PVC membrane changes after the addition of PF127 as the additive in forming the membrane pores ^9^.

As shown in Figure 3a, the original PVC membrane has an upper layer that is thicker than the upper layer structure after the addition of PF127. The exchange of solvent from the polymer solution to the coagulation bath occur slowly in the case of the original PVC membrane, and contribute to the formation of a thick upper layer that is larger than in the other systems ^15^. After the addition of PF127, the membrane surface becomes more hydrophilic and the affinity between the casting solution and water increases, so the polymer solution will attract more water and the diffusion process of water into the polymer matrix will be faster ^16^. As a consequence of this mechanism, large macrovoids and a thin upper layer are formed. As can be seen in crosssection layer, the finger like structure forms at PVC membrane with 3wt% of PF127 is dominate a half of the crossection area of the membrane. While, in case of the addition of 5 and 7wt% of PF127, the finger-like structure formed in all cross-section area of the membrane. In other words, the increase in the PF127 concentration results in larger pores and a thinner upper layer of the membrane.

### Membrane hydrophilicity

**Figure 4.**
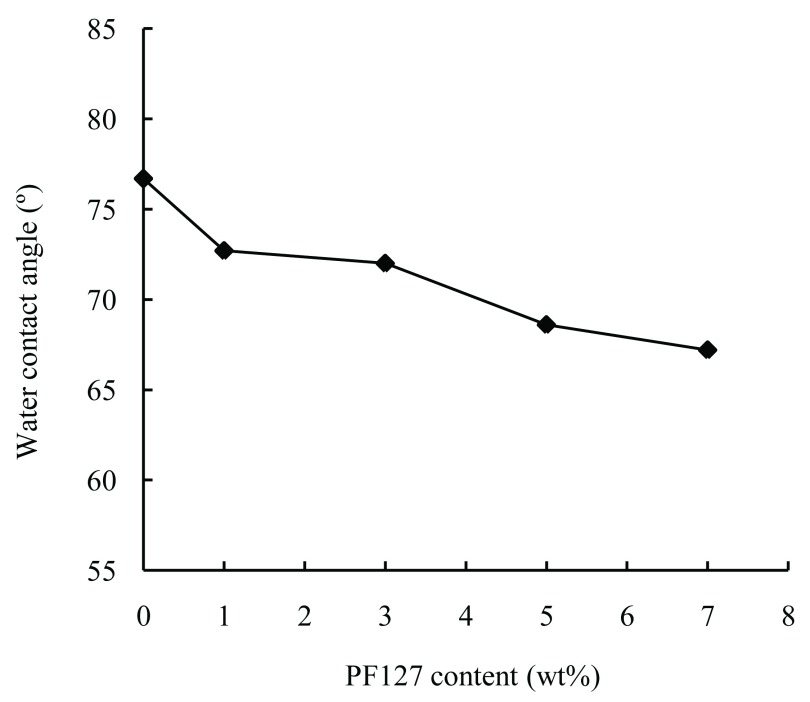
The value of the water contact angle of the PVC membrane based on the concentration of PF127 (n=10 per PF127 concentration).

The measurement of the water contact angle is the simplest way to identify the degree of hydrophilicity and hydrophobicity of the membrane ^15^. The hydrophilicity of the membrane, as measured by the water contact angle meter, is shown in Figure 4. The addition of PF127 is proven to improve the hydrophilicity of the membrane, as indicated by the decrease in the water contact angle. The existing PEO segment contained in the PF127 on the membrane surface contributed to the improvement in the membrane hydrophilicity. The hydrophilic nature of PEO chain in Pluronic increased the diffusion rate of non-solvent during membrane solidification. The rapid diffusion of nonsolvent promoted instantaneous demixing, which enhanced macrovoid formation. It has been reported that a rapid precipitation caused by the hydrophilicity of the additive results in higher surface porosity and more porous sublayer, leading to a higher water permeation ^17^. A number of studies on the mechanism of the decreased water contact angle of various membrane modifications with Pluronic have been reported by researchers ^17– 20^. The most hydrophilic membrane surface obtained in this study was found in the PVC membrane with the addition of 7wt% additive, with a contact angle of 67.2°.

### Filtration performance

**Figure 5.**
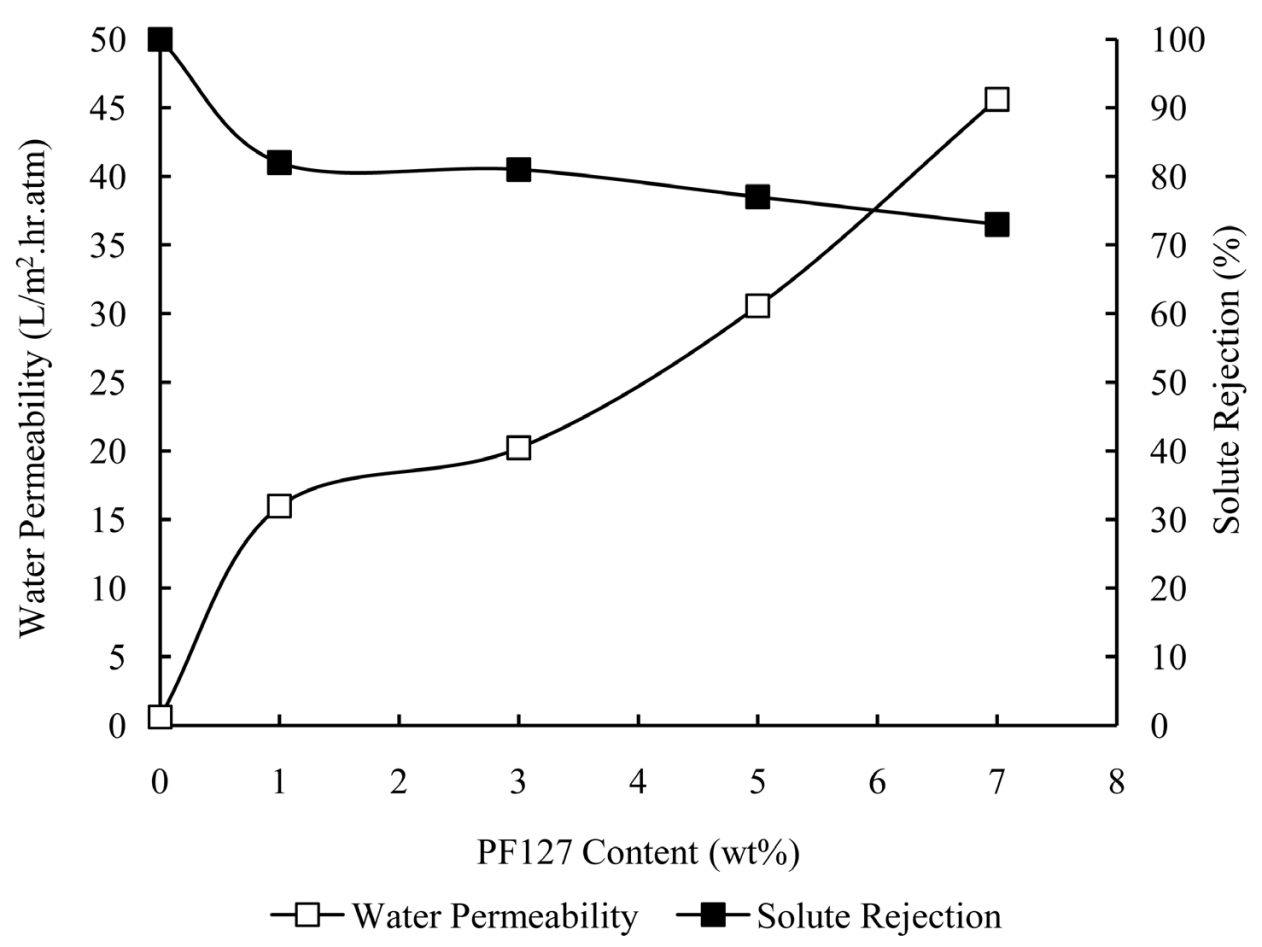
Pure water permeability and solute rejection in PVC membrane based on PF127 concentration (n=4 for permeability, n=3 for solute rejection per PF127 concentration).

The water permeability and rejection profile of the original PVC and PVC blend membrane are shown in Figure 5. The original PVC membrane has a water permeability of about 0.616 l/m ^2^.h.atm. After the addition of 1 wt% of PF127 to the dope solution additive, the water permeability increases significantly. The PEO chain in PF127 increases the pore size of membrane. Therefore, the amount of water that passes through the membrane is higher than that of the membrane without the polymeric additive. The change in the bottom layer structure of the PVC blend membrane is also evidence of the increased water permeability ( Figure 3). As reported by many authors, the addition of an appropriate amount of hydrophilic polymer to the dope solution might enhance the membrane pores ^7, 15, 21, 22^, and, consequently, high permeation would be obtained. In this work, the highest water permeability reached 45.618l/m ^2^.h.atm, which was obtained in the case of the blend membrane with the PF127 concentration of 7wt%.

Figure 5 also shows the rejection efficiency of the dextran solution. The original PVC membrane is able to reject the dextran molecules by up to 100%. The addition of PF127 at a high concentration causes a decline in the rejection efficiency. The solution sample for the rejection experiment was prepared by dissolving a low molecular weight of dextran (i.e., 10.000 Da). This may be the reason for the reduction of rejection efficiency at high concentration of additive in this work. To achieve the best performance for permeation and selectivity, the optimization of the polymer solution could be designed by changing the relative composition of the PVC and the PF127.

### Membrane shrinkage

In reference to the separation industry, membranes are expected to sustain in a wide range of temperature conditions. To determine the resistance of PVC/PF127 membranes in high-temperature conditions, a shrinkage test was performed by drying the membranes at 80°C; the results are shown in Table 1. The original PVC membrane did not suffer significant shrinkage after exposure to a temperature of 80°C, nor did the blending of PF127 into a polymer solution contribute seriously to the shrinkage of the PVC membrane. As shown in Table 1, an increase in the additive concentration of up to 7 wt% only has a small impact on the decrease in membrane size. PVC is one of the most widely used polymers in UF membranes, owing to its excellent physical and chemical properties. PVC polymer has a melting point of 212°C, and material fabricated from this polymer can only be degraded at high temperature ^23^.

**Table 1.** Shrinkage observation of PVC membrane (n=3 per PF127 concentration).

PF127 in PVC membrane (wt%)	Shrinkage (%)
0	0.67
1	2.07
3	3.33
5	5.00
7	9.33

Raw data for water permeability and solute rejection Click here for additional data file. Copyright: © 2018 Arahman N et al. 2018 Data associated with the article are available under the terms of the Creative Commons Zero "No rights reserved" data waiver (CC0 1.0 Public domain dedication).

Raw data for water contact angle Click here for additional data file. Copyright: © 2018 Arahman N et al. 2018 Data associated with the article are available under the terms of the Creative Commons Zero "No rights reserved" data waiver (CC0 1.0 Public domain dedication).

Raw data for membrane shrinkage test Click here for additional data file. Copyright: © 2018 Arahman N et al. 2018 Data associated with the article are available under the terms of the Creative Commons Zero "No rights reserved" data waiver (CC0 1.0 Public domain dedication).

## Conclusion

The fabrication of PVC membrane with PF127 as an additive has been performed in this work. The characteristics and performance of the membrane have been analyzed in terms of the morphology, hydrophilic or hydrophobic properties, water permeability, and solute rejection, as well as membrane shrinkage. From this recent study, it can be concluded as follows:

1. Morphological analysis using SEM shows the increasing the membrane porosity after addition of PF127.

2. The water permeability of PVC membrane increases from 0.61 to 45.61 l/m ^2^.hr.atm after addition of 7wt% PF127. However, the optimum filtration result, water permeability and rejection are found on the membrane with 3wt% addition PF127. It reach 20,2 L/m ^2^.h for permeability and 68,66% for solute rejection.

3. The number and length of the macrovoid structure in the center section of the membrane increased and change after the presence of PF127

4. Regarding the water contact angle observation, it is found that the hydrophilicity of the membrane improves as the proportion of PF127 is increased.

5. On the basis of the results of the shrinkage test, it can be concluded that the PVC membrane obtained in this research is able to withstand extreme temperature conditions of up to 80°C.

6. Regarding the experimental results, it can be concluded that PF127 succeeded in improving hydrophilic properties, filtration performance, and maintaining the stability of the membrane. Thus, the PVC-FP127 is useful to be applied in the water treatment industry.

## Data availability

The data referenced by this article are under copyright with the following copyright statement: Copyright: © 2018 Arahman N et al.

Data associated with the article are available under the terms of the Creative Commons Zero "No rights reserved" data waiver (CC0 1.0 Public domain dedication). 

**Dataset 1. Raw data for water permeability and solute rejection**. DOI: 10.5256/f1000research.15077.d206401 ^24^.

**Dataset 2. Raw data for water contact angle**. DOI: 10.5256/f1000research.15077.d206402 ^25^.

**Dataset 3. Raw data for membrane shrinkage test**. DOI: 10.5256/f1000research.15077.d206403 ^26^.
