# Supplementary material for: Morphology and performance of polyvinyl chloride membrane modified with Pluronic F127
Source: F1000Res. 2018 Jul 10;7:726. Originally published 2018 Jun 12. [Version 2] doi: 10.12688/f1000research.15077.2 (PMC6069734; doi:10.12688/f1000research.15077.2)
Supplement: Raw data for water permeability and solute rejection [file f1000research-7-16896-s0000.tgz › 911ea52d-b232-49ea-8e10-740e5845ebf3_1-_Water_permeability_and_solute_rejection.doc]

1. Water Permeability dependent of Pluronic F127 content

| Membrane Type | Water Permeability  (L/m^2^.hr.atm) | Water Permeability (averages)  (L/m^2^.hr.atm) |
| --- | --- | --- |
| PVC15wt%+DMAC87wt%  (Original) | 0.627395  0.565022  0.633021  0.637301 | 0.615685 |
| PVC15wt%+F127-1wt%-DMAC86wt% | 16.75499  14.68814  16.19242  16.21688 | 15.96311 |
| PVC15wt%+F127-3wt%-DMAC84wt% | 19.8247  19.39666  20.59519  21.02324 | 20.20995 |
| PVC15wt%+F127-5wt%-DMAC82wt% | 32.59274  33.09417  28.22666  28.33673 | 30.56258 |
| PVC15wt%+F127-7wt%-DMAC80wt% | 48.23481  46.42479  43.77089  44.03995 | 45.61761 |

2. Solute rejection of the membrane dependent of Pluronic F127 content

| Membrane | Solute Rejection (%) | Solute Rejection ; Averages  (%) |
| --- | --- | --- |
| Original | 100  100  100 | 100 |
| 1wt % F127 | 80  85  80 | 82 |
| 3wt % F127 | 80.50  78.66  83.66 | 81 |
| 5wt % F127 | 75.88  78.50  76.66 | 77 |
| 7wt % F127 | 70.50  73.60  75.66 | 73 |
