# Supplementary material for: Morphology and performance of polyvinyl chloride membrane modified with Pluronic F127
Source: F1000Res. 2018 Jul 10;7:726. Originally published 2018 Jun 12. [Version 2] doi: 10.12688/f1000research.15077.2 (PMC6069734; doi:10.12688/f1000research.15077.2)
Supplement: Raw data for water contact angle [file f1000research-7-16896-s0001.tgz › 905e178a-7bf6-48dc-9108-9e2ebef11684_2-_Water_contact_angle.doc]

3. Water Contact Angel data measurement of membranes

| Membrane Sample | 1 | 2 | 3 | 4 | 5 | 6 | 7 | 8 | 9 | 10 | Avg. |
| --- | --- | --- | --- | --- | --- | --- | --- | --- | --- | --- | --- |
|  | C.A.(ﾟ) | C.A.(ﾟ) | C.A.(ﾟ) | C.A.(ﾟ) | C.A.(ﾟ) | C.A.(ﾟ) | C.A.(ﾟ) | C.A.(ﾟ) | C.A.(ﾟ) | C.A.(ﾟ) | C.A.(ﾟ) |
| Original membrane | 77.1 | 75.2 | 77.1 | 75.5 | 76.5 | 75.9 | 76.2 | 74.4 | 76.8 | 79.4 | 76.7 |
| Pluronic F127 1wt% | 68.4 | 70.5 | 71.7 | 72.2 | 73.1 | 74.6 | 75.5 | 72.2 | 74.6 | 73.6 | 72.7 |
| Pluronic F127 3wt% | 72.4 | 72.4 | 70.4 | 73.6 | 72.5 | 72.0 | 73.4 | 69.5 | 71.7 | 72.4 | 72.0 |
| Pluronic F127 5wt% | 68.7 | 68.7 | 69.3 | 65.8 | 68.1 | 68.0 | 70.9 | 65.00 | 70.3 | 71.4 | 68.6 |
| Pluronic F127 7wt% | 68.9 | 67.2 | 67.7 | 66.6 | 67.2 | 68.3 | 66.0 | 65.9 | 67.2 | 66.6 | 67.2 |
