# Supplementary material for: Morphology and performance of polyvinyl chloride membrane modified with Pluronic F127
Source: F1000Res. 2018 Jul 10;7:726. Originally published 2018 Jun 12. [Version 2] doi: 10.12688/f1000research.15077.2 (PMC6069734; doi:10.12688/f1000research.15077.2)
Supplement: Raw data for membrane shrinkage test [file f1000research-7-16896-s0002.tgz › 86acf59f-4fd0-4611-b5f4-615b2689d3d5_3-_Shrinkage_test.doc]

1. **Membrane srink.**

Water uptake = $\frac{L_{0}-L_{1}}{L_{0}}x100\%$

L_0_	= Length of wet membrane (cm)

L_1_	= Length of dried membrane (cm) (100^o^C, 24 hrs)

L_2_	= Length of membrane after immersed in hot water (80^o^, 1 hr)

L_3_	= Length of membane after dried in vacum oven (100^o^C, 24 hrs)

| Membrane type | L_0_ | L_1_ | Membrane srink  (%) |
| --- | --- | --- | --- |
| 1 | 50.0  49.5 | 47.5  47.0 | 5.00  5.00 |
| 2 | 50.0  50.0 | 35.0  32.0 | 30.00  36.00 |
| 3 | 50.0  50.0 | 30.0  32.0 | 40.00  36.00 |
|  |  |  |  |
| 4 | 50.0  50.0 | 47.5  48.0 | 5.00  4.00 |
| 5 | failed | failed | failed |
| 6 | 50.0  50.0 | 35.0  35.0 | 30.00  30.00 |
|  |  |  |  |
| 7 | 50.0  50.0 | 42.0  41.0 | 16.00  18.00 |
| 8 | 50.0  50.0 | 29.0  25.0 | 42.00  50.00 |
| 9 | 50.0  50.0 | 36.0  32.0 | 28.00  36.00 |
