# Supplementary material for: Morphology and performance of polyvinyl chloride membrane modified with Pluronic F127
Source: F1000Res. 2018 Jul 10;7:726. Originally published 2018 Jun 12. [Version 2] doi: 10.12688/f1000research.15077.2 (PMC6069734; doi:10.12688/f1000research.15077.2)
Supplement: Peer review history

# Peer review: Referee response for version 2

**DOI:** 10.5256/f1000research.16896.r35902

**Published:** 2018-07-30

**Authors:** Zulfan Adi Putra

**Affiliations:** 1 Chemical Engineering Department, Universiti Teknologi Petronas, Bandar Seri Iskandar, Malaysia

The authors have corrected the paper accordingly. Herewith I recommend accepting this paper.

I have read this submission. I believe that I have an appropriate level of expertise to confirm that it is of an acceptable scientific standard.

# Peer review: Referee response for version 2

**DOI:** 10.5256/f1000research.16896.r35903

**Published:** 2018-07-11

**Authors:** Heba Abdallah

**Affiliations:** 1 Chemical Engineering and Pilot Plant Department, National Research Centre, Giza, Egypt

I recommend accepting the article

I have read this submission. I believe that I have an appropriate level of expertise to confirm that it is of an acceptable scientific standard.

# Peer review: Referee response for version 1

**DOI:** 10.5256/f1000research.16418.r34922

**Published:** 2018-06-22

**Authors:** Heba Abdallah

**Affiliations:** 1 Chemical Engineering and Pilot Plant Department, National Research Centre, Giza, Egypt

The article studied the effect of addition of pluronic F127 on the PVC membrane morphology and performance.

 The article needs Minor Revision.

**Introduction**

 The authors should do a comparison between this work and other previous work in terms of different surfactant additions, rejection % and permeability.

**In section membrane preparation**

- I think this is wrong - thickness in micrometer (0.450 µm too thin) the wet thickness using most knifes or applicators can reach to 0.45 mm or 450 micrometer
- Explain where PF127 is added - is it added in polymeric solution or in coagulation bath?

 

**SEM section**

 SEM section need more discussion, explain why the addition of 3% PF127 in fig 2b indicates the biggest finger like formation compared to 5 and 7%?

**Membrane hydrophilicity**

 Explain how the PEO effects the membrane, with a reference?

**Membrane Shrinkage**

 The method of measuring shrinkage is not correct, it depends on the length of wet membrane, which means the blend membrane will absorb more water due to hydrophilicity, so I think that can affect the wet length of the membrane.

 See this reference and repeat your shrinkage experiment:

 Bilad M *et al*, 2015 ^1^

**Conclusion**

 Re-write the conclusion in points to indicate your results clearly.

I have read this submission. I believe that I have an appropriate level of expertise to confirm that it is of an acceptable scientific standard.

## References

[1] BiladMGuillen-BurriezaEMavukkandyMAl MarzooqiFArafatH: Shrinkage, defect and membrane distillation performance of composite PVDF membranes. *Desalination*.2015;376: 10.1016/j.desal.2015.08.015 62-72 10.1016/j.desal.2015.08.015

# Response

**Published:** 2018-07-02

**Authors:** Nasrul Arahman

**Affiliations:** Syiah Kuala Uiversity, Indonesia

1. **Heba Abdallah**, Chemical Engineering and Pilot Plant Department, National Research Centre, Giza, Egypt 

 

*Comment 1*

 The authors should do a comparison between this work and other previous work in terms of different surfactant additions, rejection % and permeability.

*Response*

 F The comparison between previous works has been added to Introduction at third paragraph. The manuscript is written as follows:

 The hydrophilic polymers that are frequently used as an additive are polyvinylpyrrolidone, polyethylene glycol, Brij, Tetronic, and Pluronic ^7^ ^, 8^. Of these, Pluronic is used as a surface modifying agent for many hydrophobic polymers. Raslan and Mohammad ^9^ added Pluronic F127 to a polysulfone membrane. The resulting membrane is resistant to fouling and possesses good pore distribution ^9^ . The highest flux reached up to around 800 L/m ^2^.h at 4,5 bar with 4,8 g addition of PF127. Pluronic has also been used to improve the anti-fouling of cellulose acetate (CA) membranes ^10^ . The combination of CA polymer and Pluronic surfactant results in a membrane that is more resistant to fouling and has a more stable filtration profile. Hydrophilic surfactant Tween20 and Tween80 has been used to enhance the permeation and antifouling properties of PVC membrane. The surfactants were added as 0; 1; 3; 5; and 7 % to dope membrane. The result showed that higher concentration of the both surfactant resulting higher water flux. The highest water flux is 328,6 L/m2.h with 7% Tween20 addition. Contrary to the rejection performance of BSA, the result showed decreasing rejection value in higher surfactant addition. The BSA rejection by PVC original membrane is about 97%, after addition of surfactant additives the rejection value decreased up to around 86-87,5%  at 7% Tween20 and Tween80.

*Comment 2*

- I think this is wrong - thickness in micrometer (0.450 µm too thin) the wet thickness using most knifes or applicators can reach to 0.45 mm or 450 micrometer

 

- F There was a typo and it has been revised in membrane preparation section as follows:

The solution was then framed on a glass plate using an automatically adjustable applicator (YBA-3, Yoshimitsu, Japan) at a thickness of 450 µm.

*Comment 3*

- Explain where PF127 is added - is it added in polymeric solution or in coagulation bath?

 

 Response

 Fwe have rewrite the script in membrane preparation section

 To improve the performance of the membrane, PF127 was added at concentrations of 1, 3, 5 and 7 wt%  to polymeric solutions and dissolve until homogene.

*Comment 4*

 SEM section need more discussion, explain why the addition of 3% PF127 in fig 2b indicates the biggest finger like formation compared to 5 and 7%?

 Response

 F The more discussion of finger like formation has been added in the second paragraph of membrane morphology discussion.

 The finger like structure forms at PVC membrane with 3wt% of PF127 is dominate a half of the crossection area of the membrane. While, in case of the addition of 5 and 7wt% of PF127, the finger-like structure formed in all cross-section area of the membrane

*Comment 5*

 Explain how the PEO effects the membrane, with a reference?

 Response

 F the more discussion of PEO effects has been written in membrane hydrophilicity section.

 the hydrophilic nature of PEO chain in Pluronic increased the diffusion rate of non-solvent during membrane formation. The increasing of nonsolvent in-diffusion rate promoted instantaneous demixing, which enhanced macrovoid formation. It has been reported that a rapid precipitation caused by the hydrophilicity of the additive results in higher surface porosity and more porous sublayer, leading to a higher water permeation.

 C.H. Loh et al. / Journal of Membrane Science *380 (2011) 114–123*

   

*Comment 6*

 The method of measuring shrinkage is not correct, it depends on the length of wet membrane, which means the blend membrane will absorb more water due to hydrophilicity, so I think that can affect the wet length of the membrane.

 See this reference and repeat your shrinkage experiment:

 Bilad M *et al*, 2015 ^1^

 Response

**F** Thank you for your correction, we agree with your idea that the length of wet membrane has an effect on membrane shrinkage. We, therefore, calculated the membrane shrinkage by measuring the length of the wet cast and the dried membrane. Research group of Bilad (Bilad et al. 2015) designed the shrinkage calculation by measuring of the width of wet cast and the dried membrane.

 We revised the manuscript as follow:

 To study the effect of the blending of pluronic additives on the toughness of the PVC/ PF127 membrane, a membrane shrinkage test was performed. The wet cast of membrane at each PF127 concentration was dried in the oven for 24 hours at 80°C. The shrinkage of the membrane was calculated using Equation (3).

 Membrane shrink (%) =  ((L0-L1)/(L0)) x 100

 Where L _0_ = length of wet membrane (cm) and L _1_ = length of dried membrane (cm).

*Comment 7*

 Re-write the conclusion in points to indicate your results clearly.

 Response

 F the conclussion in points has been re-written.

 The fabrication of PVC membrane with PF127 as an additive has been performed in this work. The characteristics and performance of the membrane have been analyzed in terms of the morphology, hydrophilic or hydrophobic properties, water permeability, and solute rejection, as well as membrane shrinkage. From this recent study, it can be concluded as follows:

1. Morphological analysis using SEM shows the increasing the membrane porosity after addition of PF127.
2. The water permeability of PVC membrane increases from 0.61 to 45.61 l/m2.hr.atm after addition of 7% PF127. However, the optimum filtration result, water permeability and rejection are found on the membrane with 3% addition PF127. It reach 20,2 L/m2.h for permeability and 68,66% for solute rejection.
3. The number and length of the macrovoid structure in the center section of the membrane increased and change after the presence of PF127
4. Regarding the water contact angle observation, it is found that the hydrophilicity of the membrane improves as the proportion of PF127 is increased.
5. On the basis of the results of the shrinkage test, it can be concluded that the PVC membrane obtained in this research is able to withstand extreme temperature conditions of up to 80°C.
6. Regarding the experimental results, it can be concluded that PF127 succeeded in improving hydrophilic properties, filtration performance, and maintaining the stability of the membrane. Thus, the PVC-FP127 is useful to be applied in the water treatment industry.

# Peer review: Referee response for version 1

**DOI:** 10.5256/f1000research.16418.r34924

**Published:** 2018-06-21

**Authors:** Zulfan Adi Putra

**Affiliations:** 1 Chemical Engineering Department, Universiti Teknologi Petronas, Bandar Seri Iskandar, Malaysia

The work is about adding PF127 as the additive of a PVC membrane. Membrane characteristics and performances are measured. 

 It is, however, not clear why the addition is only until 7%. Is there any reason for this? Looking at the results, better performance could be obtain for higher concentration of PF127. In this sense, we cannot conclude the way it is concluded now. 

 The same goes with the shrinkage test, where it is only tested at 80 degC. Hence, the respective conclusion is not correct. At 7%, the shrinkage is about 10%, is it not already significant shrinkage?

 For the permeability test, it will be clearer if all various additives are put on the same graph, with the x-axis as the time and y-axis as the permeability. Then, we can see and compare the performance better. 

 All in all, the analysis of the data and the conclusion need to be revisited.

I have read this submission. I believe that I have an appropriate level of expertise to confirm that it is of an acceptable scientific standard, however I have significant reservations, as outlined above.

# Response

**Published:** 2018-07-02

**Authors:** Nasrul Arahman

**Affiliations:** Syiah Kuala Uiversity, Indonesia

*Comment 1*

 It is, however, not clear why the addition is only until 7%. Is there any reason for this? Looking at the results, better performance could be obtain for higher concentration of PF127. In this sense, we cannot conclude the way it is concluded now. 

 Response

 FIn this experiment, the best membrane is the membrane which has good both of permeability and selectivity. In other words, it has optimum filtration performance. As we know, permeability of membrane has opposite relation to its selectivity. Higher water permeability leads lower selectivity, therefore the optimum value of permeability and selectivity is the best performance of membrane. In this study, the addition of 7% PF127 produced highest water permeability, but it has the lowest rejection. In previous study, Lv (2007) investigated the addition of Pluronic F127 to CA membrane with the concentration 0 ; 4 ; 8 ; 12 ; 16 ; and 20 %. The result showed that the addition of PF127 higher than 8% enhance membrane porosity and decrease solute rejection. So that, in this study the use of PF127 is maximum  7%.

 The source of previous study: https://doi.org/10.1016/j.memsci.2007.02.011

*Comment 2*

 The same goes with the shrinkage test, where it is only tested at 80 degC. Hence, the respective conclusion is not correct. At 7%, the shrinkage is about 10%, is it not already significant shrinkage?

 Response

 F Thank you for the comment. In this experiment, the shrinkage test is designed at 80 degC, and the shrinkage impact is not so significant.   

 Comment 3

 For the permeability test, it will be clearer if all various additives are put on the same graph, with the x-axis as the time and y-axis as the permeability. Then, we can see and compare the performance better. 

 Response

 Fin this experimet, the all of permeability test were conducted in certain setting time. So, For that condition, the graph will be better if consist of the value of permeability and additives concentration.

*Comment 4*

 All in all, the analysis of the data and the conclusion need to be revisited.

 Response

 F the analysis and the conclussion in points has been re-written.
